# Prevalence and Predictors of Tuberculosis Coinfection among HIV-Seropositive Patients Attending the Aminu Kano Teaching Hospital, Northern Nigeria

**DOI:** 10.2188/jea.JE20080026

**Published:** 2009-03-19

**Authors:** Zubairu Iliyasu, Musa Babashani

**Affiliations:** 1 Departments of Community Medicine, Aminu Kano Teaching Hospital and Bayero University, Kano, Nigeria; 2 Departments of Medicine, Aminu Kano Teaching Hospital and Bayero University, Kano, Nigeria

**Keywords:** tuberculosis, HIV, coinfection, prevalence, Kano, Nigeria

## Abstract

**Background:**

The HIV/AIDS epidemic has been accompanied by a severe epidemic of tuberculosis (TB), although the prevalence of coinfection is largely unknown, especially in developing countries, including Nigeria. The aim of this study was to determine the prevalence and predictors of TB coinfection among HIV-seropositive Nigerians.

**Methods:**

The case files of HIV/AIDS patients attending Aminu Kano Teaching Hospital, Nigeria from January to December 2006 were reviewed.

**Results:**

A total of 1320 HIV/AIDS patients had complete records and were reviewed, among which 138 (10.5%) were coinfected with TB (95% CI, 8.9% to 12.2%). Pulmonary TB was diagnosed in 103 (74.6%) patients, among whom only 18 (17.5%) were sputum-positive. Fifty (36.2%) coinfected patients had some type of extrapulmonary TB (EPTB); 15 had both pulmonary TB and EPTB. Among the 35 patients with EPTB only, 20 (57.1%) had abdominal TB, 5 (14.3%) had TB adenitis, 5 (14.3%) had spinal TB, 3 (8.6%) were being monitored for tuberculous meningitis, and 1 (2.9%) each had renal TB and tuberculous adrenalitis. The highest prevalence of TB, 13.7% (*n* = 28), was seen among patients aged 41–50 years. TB coinfection was significantly associated with marital status, WHO clinical stage, and CD4 count. Marital status (OR, 2.1; 95% CI, 1.28–3.59; *P* = 0.04), WHO clinical stage at presentation (4.81; 1.42–8.34; *P* = 0.001), and baseline CD4 count (2.71; 1.51–6.21; *P* = 0.02) remained significant predictors after adjustment for confounding.

**Conclusions:**

The moderately high prevalence of TB among HIV-seropositive patients underscores the urgent need for strategies that lead to rapid identification and treatment of coinfection with active or latent TB.

## INTRODUCTION

Worldwide, the HIV/AIDS epidemic has been accompanied by a severe epidemic of tuberculosis (TB).^1^ The World Health Organization (WHO) reported 8.8 million new cases of TB and 1.6 million TB-related deaths in 2005, of which 195 000 were HIV-infected patients.^2^ HIV infection is the leading risk factor for TB; HIV promotes progression of latent or recent infections of *Mycobacterium tuberculosis* to active disease, and also increases the rate of recurrence of TB. People with HIV may also be more susceptibility to TB infection.^3^

There have been several studies of the prevalence of HIV in TB patients in different parts of Nigeria, with prevalences of 6.1% in Jos,^4^ 12.7% in Ife,^5^ 19% in Maiduguri,^6^ and 28.1% in Ibadan^7^; the national median is 17.0%.^8^ Elsewhere in Africa, higher prevalences of 33.2%, 43.6%, and 57.1% were reported in Chad,^9^ Tanzania,^10^ and Ethiopia,^11^ respectively. However, reports on the prevalence of TB in HIV-infected patients in Nigeria have been limited to Ibadan and Ilorin, where the prevalence of active TB among HIV/AIDS patients was reported to be 32.8%^12^ and 40%,^13^ respectively. There has been no such report from our center. The objectives of the present study were to determine the prevalence of TB in HIV-seropositive adults treated at the Prof. S. S. Wali Virology Centre of the Aminu Kano Teaching Hospital, in Kano, northern Nigeria and to identify the factors that are associated with the development of TB. This information may assist health managers and health care providers in increasing vigilance and providing integrated services that improve outcomes for people living with HIV/AIDS.

## METHODS

### Setting

Established in 1988, the Aminu Kano Teaching Hospital began offering clinical services in 1994 at a temporary site in the Murtala Mohammed Specialist Hospital, Kano and later moved to its present, permanent site in 1997. Situated in Kano, the largest commercial center of northern Nigeria, with over 9 million people,^14^ this 500-bed hospital receives patients from Kano, and from the neighboring states of Jigawa, Katsina, Kaduna, Bauchi, and Zamfara. The majority of patients are indigenous Hausa-Fulani, although the Ibo and Yoruba ethnic groups are also well represented. Most patients are farmers, traders, businessmen, or civil servants. Five days a week, the hospital operates a multidisciplinary, specialist clinic for HIV/AIDS patients, which is located at the Prof. S. S. Wali Centre. Examinations and antiretroviral drugs are provided at no charge to all patients. Similarly, a DOTS (Directly Observed Treatment, Short-Course) clinic has been established within the same complex; it provides free antituberculosis treatment to all TB patients. The present study population comprised HIV/AIDS patients attending the Aminu Kano Teaching Hospital HIV/AIDS specialist clinic from January to December 2006.

### Study design

The study was a retrospective review of patients’ case files.

### Data collection

We reviewed the medical records of all HIV-seropositive patients who had attended the Prof. S.S. Wali Virology Centre of Aminu Kano Teaching Hospital from January to December 2006. Ethical clearance was obtained from the Aminu Kano Teaching Hospital institutional review board. At the study center, HIV infection is diagnosed using a combination of the ELISA test for HIV-1 and HIV-2 and the Western blot test. A positive result on the ELISA test is confirmed using the Western blot test. The HIV testing protocol in our center has been described elsewhere.^15^ The procedure is as follows: after obtaining informed consent, 10 mL of blood is collected from the patient. The sample is centrifuged at 3000 rpm for 10 minutes. The resulting serum is first screened for the presence of HIV antibodies using the ELISA technique with Genescreen (Sanofi, Pasteur). All initially reactive samples are retested with another ELISA test kit (Biorad, USA). Serum samples that were positive on the second ELISA are then confirmed using the Western blot (Biotech, Ireland) or Immunoconfirm (Organics, Israel) test. All patients are provided with pretest and posttest counseling. HIV-seropositive patients who attended the clinic at least once and had received a diagnosis of pulmonary or extrapulmonary TB during the initial evaluation or on subsequent visits were included in this study. A diagnosis of TB coinfection was made on the basis of the Ziehl-Neelsen staining technique of sputum, imaging study, biopsy of tissue specimen, TB culture studies, or a combination of these. Bacteriological analysis was performed in all cases. Patients that were treated for TB and declared cured before presentation were not included. Data regarding TB/HIV coinfection were extracted from case records.

A standardized form was used to collect information about age, sex, marital status, educational status, and occupation. Other variables included identification of all pulmonary TB cases diagnosed by Ziehl-Neelsen staining, TB culture studies, and image studies and all extrapulmonary TB cases diagnosed by tissue histology and/or cytology among HIV-seropositive patients. In addition, baseline CD4 count and WHO clinical stage at presentation were recorded. The time of diagnosis of TB relative to antiretroviral treatment was also noted.

### Data analysis

The data were cleaned, validated, and analyzed using EPI Info version 6 software (CDC Atlanta, Georgia, USA).^16^ Quantitative variables were summarized using range, mean, and standard deviation, or median and quartiles, as appropriate. Categorical variables were tabulated using frequencies and percentages. The Mann Whitney U test was used to compare median CD4 counts, and the chi-square test was used for testing the significance of association between categorical variables. A bivariate analysis was performed, including the calculation of crude odds ratios (ORs). Adherence to antiretroviral therapy (ART) was not analyzed because only 5 patients developed TB/HIV infection during such treatment; these cases were therefore excluded from the model. All variables that were significantly associated with TB/HIV coinfection were included in a multivariate logistic regression analysis in order to determine their independent effects. Adjusted ORs and their respective 95% confidence intervals (CI) were also obtained. The level of significance was set at *P* < 0.05.

## RESULTS

### Sociodemographic characteristics

Of the 1456 patients seen at the Prof. S. S. Wali Virology Centre during the 1-year period under review, the records of 1320 (90.7%) were available for review. Of the case files reviewed, 724 (54.8%) of the patients were males and 596 (45.2%) were females, the female-to-male ratio was thus 1:1.2. Patient age ranged from 16 to 76 years, with a mean age of 34.2 ± 7.2 years. The average age [± SD] of the male and female patients was 36.0 ± 8.3 and 30.1 ± 5.2 years, respectively. More than 72% of the patients were aged between 21 and 40 years. The majority (74.6%) of patients were Muslims, who mostly (66.8%) belonged to the Hausa-Fulani ethnic groups. Three hundred and thirty-three patients (25.2%) were employed privately or by the government; 676 (51.2%) were unemployed. The remaining 311 (23.6%) were self-employed and engaged in different types of work. A total of 451 (34.2%) of the patients had a tertiary education, 285 (21.6%) had a secondary education, 185 (14.0%) had a primary education, and 399 (30.2%) had no formal education. A total of 884 (67.0%) were married, 92 (7.0%) were widowed, 229 (17.3%) were single, and 115 (8.7%) were divorced. Their sociodemographic characteristics are shown in Table 1.

**Table 1.** Sociodemographic characteristics of patients receiving antiretroviral treatment at Aminu Kano Teaching Hospital, Kano, Nigeria (*n* = 1320)

Characteristic	Males	Females	Frequency No. (%)
Age			
≤20	40 (5.5)	23 (3.9)	63 (4.8)
21–30	310 (42.9)	134 (22.5)	444 (33.6)
31–40	199 (27.5)	316 (53.0)	515 (39.0)
41–50	108 (14.9)	96 (16.1)	204 (15.5)
51–60	58 (8.0)	21 (3.5)	79 (6.0)
>60	9 (1.2)	6 (1.0)	15 (1.1)
Total	724 (100.0)	596 (100.0)	1320 (100.0)
Marital status			
Single	167 (23.1)	62 (10.4)	229 (17.3)
Married	478 (66.0)	406 (68.1)	884 (67.0)
Widowed	40 (5.5)	52 (8.7)	92 (7.0)
Divorced	39 (5.4)	76 (12.8)	115 (8.7)
Total	724 (100.0)	596 (100.0)	1320 (100.0)
Educational status			
Non-formal	112 (15.5)	287 (48.2)	399 (30.2)
Primary	87 (12.0)	98 (16.4)	185 (14.0)
Secondary	159 (21.9)	126 (21.1)	285 (21.6)
Tertiary	366 (50.6)	85 (14.3)	451 (34.2)
Total	724 (100.0)	596 (100.0)	1320 (100.0)
Religion			
Muslim	605 (83.6)	380 (63.8)	985 (74.6)
Christian	119 (16.4)	216 (36.2)	335 (25.4)
Total	724 (100.0)	596 (100.0)	1320 (100.0)
Ethnicity			
Hausa	307 (42.4)	263 (44.1)	570 (43.2)
Fulani	163 (22.5)	149 (25.0)	312 (23.6)
Igbo	81 (11.2)	68 (11.4)	149 (11.3)
Yoruba	47 (6.5)	41 (6.9)	88 (6.7)
Others	126 (17.4)	75 (12.6)	201 (15.2)
Total	724 (100.0)	596 (100.0)	1320 (100.0)

### Clinical characteristics

At presentation, 389 (29.5%), 213 (16.1%), 275 (20.8%), and 443 (33.6%) of the patients were in WHO stage I, II, III, and IV, respectively. The median baseline CD4 count was 242 lymphocytes/µL with lower and upper quartiles of 130 and 401 lymphocytes/µL, respectively. Approximately 37.2% of the patients had fewer than 200 CD4 lymphocytes/µL, 50.0% had between 200 and 599, and 12.9% had 600 or more lymphocytes/µL at baseline.

### Prevalence and pattern of TB/HIV coinfection

Of the 1320 HIV-seropositive patients, 138 were coinfected with TB: a period prevalence of 10.5% (95% CI, 8.9%–12.2%). Among these 138 patients, TB coinfection had been diagnosed before commencement of ART in 133 (96.4%); it was diagnosed during ART in 5 (3.6%). Among patients coinfected with TB/HIV, 103 (74.6%) had pulmonary TB (PTB), among whom only 18 (17.5%) were sputum-positive. The great majority, 85 (82.5%), were sputum-negative. Fifty (36.2%) coinfected patients had some type of extrapulmonary TB (EPTB); 15 had both PTB and EPTB. Among those with EPTB only, 20 (57.1%) had abdominal TB—defined as infection of the peritoneum, or the hollow or solid abdominal organs, with *Mycobacterium tuberculosis,* as confirmed by histologic examination of biopsy specimens or culture—5 (14.3%) had TB adenitis, 5 (14.3%) spinal TB, 3 (8.6%) were being followed for tuberculous meningitis, and 1 (2.9%) each had renal TB and tuberculous adrenalitis, as shown in Table 2. In addition, among the patients with EPTB, 12 had pleurisy and 3 had pericarditis.

**Table 2.** Tuberculosis presentation in TB/HIV coinfected patients at Aminu Kano Teaching Hospital, Kano

TB Type	Males	Females	Total No. (%)
PTB and/or EPTB	81 (58.7)	57 (41.3)	138 (100.0)
PTB			
Sputum-positive	10 (15.9)	8 (20.0)	18 (17.5)
Sputum-negative	53 (84.1)	32 (80.0)	85 (82.5)
Total	63 (100.0)	40 (100.0)	103 (100.0)
EPTB			
Abdominal TB	8 (44.4)	12 (70.6)	20 (57.1)
Spinal TB	4 (22.2)	1 (5.8)	5 (14.3)
TB adenitis	3 (16.6)	2 (11.8)	5 (14.3)
TB meningitis on follow-up	1 (5.6)	2 (11.8)	3 (8.6)
TB adrenalitis	1 (5.6)	—	1 (2.9)
Renal TB	1 (5.6)	—	1 (2.9)
Total	18 (100.0)	17 (100.0)	35 (100.0)

TB = Tuberculosis PTB = Pulmonary tuberculosis EPTB = Extrapulmonary tuberculosis

By sex, 81 (11.2%) of the 724 males had TB, as compared to 57 (9.6%) of the 596 females. This difference was not statistically significant (χ^2^ = 0.92, *P* = 0.34). With respect to age, the highest prevalence of TB (13.7%, *n* = 28) was observed among patients aged 41–50 years, followed by those aged 31–40 years (11.8%, *n* = 61) and 51–60 years (10.1%, *n* = 8), as shown in Table 3. This trend was not statistically significant (χ^2^_trend_ = 3.62, *P* = 0.06). With respect to educational attainment, the highest prevalence was observed among patients with a primary education (12.4%, *n* = 23), followed by those with a tertiary education (10.6%, *n* = 48) and secondary education (9.8%, *n* = 28). The prevalence of TB among patients without formal education was 9.8% (*n* = 39). Education status was not significantly associated with TB coinfection. With respect to marital status, widowed patients had the highest prevalence (17.4%, *n* = 20), followed by married patients (10.5%, *n* = 93) and single patients (7.9%, *n* = 18). Differences due to marital status were statistically significant (χ^2^ = 10.86, *P* = 0.01). HIV-seropositive patients coinfected with TB had a median CD4 lymphocyte count of 184/µL, as compared to 260/µL for those without coinfection. This difference was statistically significant (*P* < 0.05, Mann Whitney U test). With respect to WHO clinical stage, the likelihood of TB coinfection increased significantly with a higher WHO HIV stage at presentation (χ^2^_trend_ = 54.1, *P* < 0.01). The prevalences of TB coinfection in patients at WHO clinical stages III and IV were 18.5% and 15.6%, respectively.

**Table 3.** TB/HIV coinfection among HIV-seropositive patients attending Aminu Kano Teaching Hospital, Kano, Nigeria

		Frequency No. (%)			
Characteristic	Coinfected	Not Coinfected	Total	χ^2^	*P* value
Sex					
Female	57 (9.6)	539 (90.4)	596 (100.0)		
Male	81 (11.2)	643 (88.8)	724 (100.0)		
Total	138 (10.5)	1182 (89.5)	1320 (100.0)	1.49	0.47
Age (yrs)					
≤20	4 (6.3)	59 (93.7)	63 (100.0)		
21–30	36 (8.1)	408 (91.9)	444 (100.0)		
31–40	61 (11.8)	454 (88.2)	515 (100.0)		
41–50	28 (13.7)	176 (86.3)	204 (100.0)		
51–60	8 (10.1)	71 (89.9)	79 (100.0)		
>60	1 (6.7)	14 (93.3)	15 (100.0)		
Total	138 (10.5)	1182 (89.5)	1320 (100.0)	3.62*	0.06
Educational status					
Non-formal	39 (9.8)	360 (90.2)	399 (100.0)		
Primary	23 (12.4)	162 (87.6)	185 (100.0)		
Secondary	28 (9.8)	257 (90.2)	285 (100.0)		
Tertiary	48 (10.6)	403 (89.4)	451 (100.0)		
Total	138 (10.5)	1182 (89.5)	1320 (100.0)	1.91	0.59
Marital status					
Single	18 (7.9)	211 (92.1)	229 (100.0)		
Married	93 (10.5)	791 (89.5)	884 (100.0)		
Divorced	7 (7.6)	85 (92.4)	92 (100.0)		
Widowed	20 (17.4)	95 (82.6)	115 (100.0)		
Total	138 (10.5)	1182 (89.5)	1320 (100.0)	10.86	<0.01
WHO stage					
I	8 (2.1)	381 (97.9)	389 (100.0)		
II	10 (4.7)	203 (95.3)	213 (100.0)		
III	51 (18.5)	224 (81.5)	275 (100.0)		
IV	69 (15.6)	374 (84.4)	443 (100.0)		
Total	138 (10.5)	1182 (89.5)	1320 (100.0)	54.1*	<0.01
CD4 count					
<200	68 (13.8)	423 (86.2)	491 (100.0)		
200–399	37 (9.3)	359 (90.7)	396 (100.0)		
400–599	25 (8.9)	238 (91.1)	263 (100.0)		
≥600	8 (4.7)	162 (95.3)	170 (100.0)		
Total	138 (10.5)	1182 (89.5)	1320 (100.0)	11.2*	<0.01

*Chi-square test for trend

Bivariate analysis of TB coinfection by sociodemographic variables, baseline CD4 count, and WHO clinical stage at presentation revealed that TB coinfection was significantly associated with marital status, WHO clinical stage, and CD4 count. These 3 factors remained significant predictors of TB coinfection after exclusion of patients who developed TB coinfection while on ART treatment, and after adjusting for confounding by using multivariate analysis. There was a doubling of risk for TB coinfection among patients who were widowed, as compared to single patients. There was also a more than 4-fold likelihood of TB coinfection among patients presenting with WHO stage IV disease, as compared to those presenting with stage I disease. In addition, the risk of TB coinfection among patients with a CD4 count lower than 200 lymphocytes/µL was more than double that that of patients with a count higher than 600 lymphocytes/µL, as shown in Table 4.

**Table 4.** Predictors of tuberculosis coinfection among patients receiving antiretroviral treatment at Aminu Kano Teaching Hospital, Kano, Nigeria

Characteristic	Crude	Adjusted (95%CI)	*P* Value
	OR	OR	
Sex			
Male	1.19 (0.82–1.73)	1.03 (0.91–1.43)	0.12
Female	Referent		
Age (yrs)			
≥30	Referent		
31–40	1.57 (1.01–2.44)	1.26 (0.35–2.03)	0.48
41–50	1.86 (1.08–3.19)	1.23 (0.65–3.29)	0.28
>50	1.24 (0.54–2.77)	1.02 (0.31–3.01)	0.45
Marital status			
Single	Referent		
Married	1.39 (0.79–2.42)	1.21 (0.34–2.16)	0.12
Divorced	0.93 (0.35–2.56)	0.97 (0.63–3.53)	0.43
Widowed	2.50 (1.19–5.14)	2.1 (1.28–3.59)	0.04
WHO stage			
I	Referent		
II	2.5 (0.83–6.63)	1.37 (0.59–4.75)	0.20
III	5.90 (4.85–25.20)	3.22 (1.67–7.20)	0.001
IV	7.40 (4.01–20.02)	4.81 (1.42–8.34)	0.001
CD4 count			
<200	3.26 (1.47–7.49)	2.71 (1.51–6.21)	0.02
200–399	2.10 (1.59–4.97)	1.87 (1.53–5.27)	0.03
400–599	2.13 (1.61–5.27)	1.63 (1.03–4.36)	0.03
≥600	Referent		

OR = Odds Ratio CI = Confidence Interval

## DISCUSSION

In the present study, 10.5% of the HIV-seropositive patients in our center had TB. This is much lower than the 40% and 32.8% prevalences of active TB reported among HIV-seropositive patients in the Nigerian cities of Ilorin^13^ and Ibadan,^12^ respectively. However, the prevalence observed in the present study is higher than that in the United Sates.^20^ Evidence from areas with high TB and HIV burdens^17^^–^^19^ indicates a high incidence of TB/HIV coinfection. The differences observed between our center and other Nigerian centers could be due to selection factors: the other studies were conducted before free antiretroviral drugs were provided in government hospitals. Antiretroviral drugs became free of charge in Nigeria in 2005, before which only those who could afford therapy went to hospitals, sometimes as a last resort, which would have resulted in a pooling of late-stage patients at these hospitals.

As the most populous country in sub-Saharan Africa, Nigeria has the highest number of TB cases of any country on the continent, about 449 558 in 2006.^21^ This high burden from TB has placed it among the top 5 of the WHO’s high-burden TB countries. Unfortunately, DOTS is available to only 75 percent of the Nigerian population. Of even greater concern, the 20% rate of detection of new TB cases under DOTS is one of the lowest in sub-Saharan Africa.^21^ Therefore, the rising prevalence of TB among HIV patients has implications for the already overburdened health systems in resource-poor settings such as ours. TB programs are often unable to manage the high number of HIV-related TB cases and ensure completion of TB therapy. Current government efforts to control both TB and HIV/AIDS will need to be augmented dramatically in order to avert a healthcare disaster. We believe that, with a current adult coinfection rate of 9.6 percent,^21^ expansion of the DOTS programs will allow for more rapid and thorough identification of persons with HIV/AIDS.

The fact that TB was diagnosed before commencement of ART in nearly all (96.4%) of the patients with TB coinfection indicates that more vigilance among physicians could increase the detection rate, thereby allowing early treatment of TB, which would improve outcomes among these patients. The majority (74.6%) of the coinfected patients presented with PTB; 85 (82.5%) were sputum-negative. This is similar to reports from Ilorin (79%)^13^ and Ibadan (78.6%).^17^ Elsewhere in Africa, however, Range and colleagues reported a higher proportion (81.2%) of pulmonary TB among HIV-seropositive patients in Tanzania.^10^ This disparity could reflect differences in the seroprevalence of HIV between these populations. The proportion of sputum-negative patients in the present study was higher than the 53%^6^ reported among TB/HIV coinfected patients in Maiduguri, Nigeria. The preponderance of such cases among coinfected patients in our study could be due to the lower rate of caseation necrosis, and the consequent lower numbers of acid-fast bacilli in the airway.^22^ HIV may also reduce the specificity of sputum microscopy by increasing the proportion of patients with non-tuberculous mycobacteria.^23^ Currently no other diagnostic tool, including sputum culture, is available that could be used affordably in resource-poor settings, where the burden of disease is greatest. Two new tests can identify *M. tuberculosis* ribosomal RNA (MTD, Gen-Probe, San Diego, Calif.) or DNA (Amplicor, Roche Molecular Systems, Branchburg, N.J.) in clinical specimens within 24 hours, but these tests are not feasible in our setting.^24^ A study conducted in Zimbabwe reported improved yield after concentrating their specimens.^25^

The proportion of extrapulmonary cases seen in the present study (25.4%) is higher than the 21.4% and 21% reported in Ibadan^7^ and Ilorin,^13^ respectively. Among the extra-pulmonary forms, abdominal TB was the most frequent, which accords with data in the literature on populations with a similar level of immunosuppression.^26^^,^^27^ The results of the present study show that, after adjustment for other variables, TB was more frequent among widows, patients presenting at late stage (WHO clinical staging), and those with a lower baseline CD4 count. The high prevalence of TB (22.6%) among widows may be related to a loss of economic support after the demise of a breadwinner, who probably died of AIDS. The present study also shows that a high CD4 count may decrease the chance of developing TB. In the medical literature, there is no clear cutoff for CD4 count above which the risk for TB development is diminished. However, there is a clear inverse correlation between CD4 count and the risk of opportunistic infections and death.^28^^,^^29^ Markowitz et al.^30^ identified 2 major risk factors for TB progression: (1) a positive result on a protein purified derivative test at baseline or during the study, which indicates the importance of the degree of previous or current exposure to *Mycobacterium tuberculosis*, and (2) a low CD4 count, which shows the role of immunodepression in the development of active TB. Although, in the present study, some WHO stage-I and -II patients were coinfected with TB—which indicates that no stage is immune to TB coinfection—a higher likelihood of TB coinfection at advanced WHO stages of the disease was clearly observed in our patients. In the present study, the highest prevalence (17.6%) of TB was seen among patients aged 41–50 years, which differs from the bimodal distribution observed in Ibadan, Nigeria,^12^ and the peak in individuals aged 20–40 years noted in Jos,^4^ Nigeria. This disparity may be due to differences in demographic characteristics and/or HIV risk distribution among different age groups in these locations.

This study had several limitations. First, this was a facility-based study; centers such as ours commonly receive the most severe, complicated cases, and the diagnostic difficulties are therefore greater. This may be an example of the iceberg phenomenon, ie, the prevalence observed in the present study may not accurately reflect that of the region as a whole. Secondly, the DOTS center at our facility became operational only in early 2007; therefore, some earlier cases may have been referred to the Infectious Diseases Hospital for supervision of DOTS therapy, which may have led to an underestimation of prevalence. Finally, because of the study design, the predictors of TB/HIV coinfection identified in the present study may not be exhaustive. A known drawback of retrospective reviews of case records is that the number of variables available for examining predictors of disease is limited. A prospective study is therefore recommended to explore other factors that may be associated with TB/HIV coinfection in this study population.

The moderately high rate of TB coinfection among patients at our center underscores the importance of informing health practitioners of the challenges of managing these patients, beyond simply treating the individual diseases. Control of both TB and HIV is likely to be most successful if programs collaborate whenever possible and are closely integrated with other healthcare providers. We therefore need to intensify our search for TB in HIV-seropositive patients who attend HIV service outlets and to provide isoniazid prophylaxis to individuals with known or suspected latent infection with *Mycobacterium tuberculosis*, in order to prevent progression to active disease after exclusion of active TB. There is a need to institute TB control measures both in healthcare settings and in places where people with TB and HIV frequently congregate. Measures should include early recognition, diagnosis, and treatment of TB suspects, particularly those with pulmonary TB; separation of pulmonary TB suspects until a diagnosis is confirmed or excluded; and maximization of natural ventilation to protect HIV-seropositive individuals from possible exposure to TB.
